# Detection of Hyaluronic Acid Receptor in Human Vocal Folds by immunohistochemistry

**DOI:** 10.1016/S1808-8694(15)31089-2

**Published:** 2015-10-19

**Authors:** Luiz Henrique Fonseca Barbosa, Hugo Valter Lisboa Ramos, Luciano Rodrigues Neves, Noemi Grigoletto de Biase, Celina Oshima, José Eduardo de Sá Pedroso, Paulo Augusto de Lima Pontes

**Affiliations:** 1 Master's degree in science, ENT Department, UNIFESP-EPM; adjunct professor of ENT, UFBA; 2 Master's degree in science, ENT Department, UNIFESP-EPM; doctoral student, ENT Department, UNIFESP-EPM; 3 Master's degree in science, ENT Department, UNIFESP-EPM; doctoral student, ENT Department, UNIFESP-EPM; 4 Doctor in medicine, ENT Department, UNIFESP-EPM; teaching professor in the ENT Department post-graduate course, UNIFESP-EPM; 5 Doctor in medicine, Pathology Department, UNIFESP-EPM; teaching professor in the molecular pathology sector, Pathology Department UNIFESP-EPM; 6 Doctor in medicine, ENT Department, UNIFESP-EPM; coordinaor of the laryngology and voice sector, ENT Department, UNIFESP-EPM; 7 Full professor, ENT Department, UNIFESP; head of the ENT Department, UNIFESP. Otorhinolaryngology and Head & Neck Surgery Department, Sao Paulo Federal University - Paulista Medical School

**Keywords:** immunohistochemistry, larynx, vocal fold, hyaluronic acid

## Abstract

Hyaluronic acid receptor is a glycoprotein of the plasmatic membrane, and the CD44 is its representative, expressed in many cell types where it has the task of cell adhesion.

**Aim:**

the goal of the present experimental study is to investigate the possibility of using immunohistochemistry to identify the distribution of hyaluronic acid along the vocal fold.

**Materials and Methods:**

We resected the normal vocal folds from a normal 23 year-old male black individual. The slides were analyzed by means of a histomorphometric study, comparing the color intensity in the superficial, middle and deep layers of the lamina propria. In the silanized slides we used immunohistochemistry, and evaluated the slides under light microscopy with 40x magnification, and the color changed to brown when there was a reaction with the receptor for hyaluronic acid.

**Results:**

Immunohistochemical findings showed the presence of hyaluronic acid receptors in the epithelium covering the vocal fold, being more concentrated in the central region of the vocal fold.

**Conclusion:**

immunohistochemistry, used to assess the distribution of hyaluronic acid receptors in the central portion of the vocal fold, proved it to be present in the vocal fold epithelium and it prevailed in its middle third.

## INTRODUCTION

Phonation is the most recent function in laryngeal phylogenesis; it uses the same anatomical structures as breathing and swallowing, but requires increased central nervous system control.

The physiology of sound production by the vocal folds (VFs) is intimately related with the features of the lamina propria (LP).

A histological description of the vocal fold (VF) structure includes the following layers: a superficial layer, also named Reinke's space, which is formed by loosely organized elastic and collagen fibers; an intermediate layer, formed by a larger amount of elastic and collagen fibers; and a deep layer in the LP, formed by a larger amount of collagen fibers laid parallel along the long axis of the VFs. The intermediate and deep layers of the LP together form the vocal ligament. The body-cover theory was developed based on the trilaminar structure of the LP; it is correlated with VF vibration according to the distribution of elastic and collagen fibers.^1^

The VF mucosa is lined by ciliated squamous pseudostratified epithelium on the vestibular and intraglottic aspects; nonkeratinizing stratified epithelium lines the free or medial contact surface.

The VF lining (formed by epithelium and the surface layer of the LP) vibrates over the deeper layers of the LP (vocal ligament and vocal muscle) forming the mucous wave of VFs.^2^

Freedom of movement of the VF lining depends on the status of the LP, which in turn depends on the structure of the extracellular matrix (ECM) and the distribution of proteins. Both affect the voice quality.

The ECM contains stable macromolecular complexes, which are formed by various structures that are produced within and exported from cells. These macromolecules modulate tissue structure, biomechanics and physiology, which are closely related with the quantity of vibrating tissue.^3^

Hyaluronic acid (HA) is a high molecular weight non-sulphated glycosaminoglycan (GAG) that is synthesized on the plasmatic membrane by an enzyme complex of various cell types; it is the main GAG in connective tissues and is released out of cells as it is produced.^4^, ^5^, ^6^

HA in VFs promotes adequate viscoelasticity of the LP so that the mucous wave may be formed during phonation. HA also cushions impact, protecting the LP from trauma caused by the constant VF contact during phonation. Additionally, HA preserves tissue viscosity and elasticity, thus maintaining the energy threshold and control of the fundamental frequency.^5^, ^6^, ^7^, ^8^

HA is distributed in the basal membrane and the vocal muscle; it is absent in the VF epithelium, as revealed in histochemical studies of rabbit larynxes by means of specific binding proteins.^9^

The HA receptor is a cell plasmatic membrane glycoprotein, the main one being the CD44 receptor.^10^ These receptor isoforms are extremely heterogeneous in the primary structure. It is believed that such a complex structure is important for its functions, such as cell-cell and cell-ECM interactions.^11^

The CD44 receptor, when acting as a cell adhesion receptor, is expressed in various cell types, including lymphocytes, myeloid cells, fibroblasts, retinal cells and glial cells of the central nervous system.^11^

HA-degrading enzymes may inhibit cell aggregation in various cells types. Studies have shown that receptors recognize the sugar sequence in HA; they may bind with low affinity to chondroitin sulphate, but not to another GAG.^10^

The HA-removing mechanism is proportional to the concentration of CD44 receptors. These receptors allow HA-binding to cell surfaces; HA may also be carried into cells, where it is degraded by lisosomal enzymes.^12^

Epithelial tissue receptors are preferentially expressed in proliferating cells.^10^

An elevated concentration of HA and CD44 is related with the genesis of tumors; CD44, therefore, is considered as a tumor marker, mostly for urologic and breast cancers.^11^

Studying the concentration of HA and its receptors across VF layers is important for understanding its functions and for comparison purposes with studies of altered VFs.

The purpose of this experimental article was to investigate the possibility of using an immunohistochemical method for identifying the distribution of HA receptors across human VFs.

## MATERIAL AND METHOD

The Research Ethics Committee analyzed and approved the research project (CEP: 1298/04); the medico-legal authorities also gave their approval. Following this process, the study was started.

Based on medico-legal guidelines, ten VFs were harvested from five male cadavers aged between 20 and 40 years; three were white and two were black. Death was by firearms. Individuals that had been subjected to tracheal intubation, that had neck trauma and over 12 hours of death, were excluded from the study.

Access was by an anterior cervicotomy, which made it possible to identify the laryngeal structure. The pharyngeal lumen was incised supraglottically, for identifying the glottic area. The larynx was dissected from neighboring structures and removed from the cadaver after being separated from the trachea, between the second and third cartilaginous ring.

The posterior larynx was incised in the interarytenoid muscles and the cricoid cartilage. A macroscopic examination was made of the exposed VFs to exclude those with injuries, hematomas, hemorrhage or other signs of trauma. VFs with no macroscopic lesions were measured and dissected from the thyroid cartilage, along the internal perichondrium. After removal, the VFs were fixed on a wood surface, preserving the anatomical distances by means of pins to avoid tissue retraction or torsion. The muscles were used as fixation points for preserving the epithelium and the LP.^13^^,^^14^

A 0.9% saline solution in individual flasks was used for transporting the VFs from the medico-legal facility to the laboratory, to avoid VF dehydration.

VFs that presented any microscopic alterations were discarded. A DF Vasconcelos 9000 microscope, at 40 times magnification, was used for this analysis. After this analysis, the VFs of a 23-year-old black individual were considered as normal and the right VF was randomly chosen for this study.

The VF was fixated in a buffered 10% formaldehyde solution. Repeated baths in pure ethyl alcohol were used for tissue dehydration. Xylol and paraffin baths were done until the VFs were paraffined.

Cross-sectional cuts were made in nine areas, in 1 mm intervals, for a complete analysis of the VF. These segments went from the anterior VF border, named region 1, to the vocal process of the arytenoid cartilage, named region ^9^. All nine segments were included in different paraffin blocks. Each block was numbered according to its position in the VF, for identification purposes.

Four 4-micrometer thick sections were made of each VF segment for the histological and immunohistochemical slides. Three sections were stained with hematoxyllin-eosin (HE), Masson's trichrome and Alcian Blue; the fourth section - in previously silanized slides - was used for the immunohistochemical study. HE and Masson's trichrome slides were used for histological orientation purposes. Polyclonal antibodies against the HA-binding protein, obtained from freeze-dried bovine nasal cartilage (manufactured by USBiological lab) were used.

A histomorphometric investigation was made of the slides; this involves comparing color intensity in the superficial, middle and deep LP layers.

The field of study in the superficial layer was the area next to the base membrane. The field of study in the middle layer was the areas next to the muscle plane; the field of study in the deep layer was the middle portion of the LP.

Optic microscopy (40 X magnification) was used in observing the silanized slides for the immunohistochemical study. Reaction with HA receptors was seen as a brown color.

Slide images were digitized using a Sony Hyper HAD digital camera coupled to an optic microscope (Laboval 4) at 400 X magnification. The DIRACOM3 software (currently renamed Imagelab 2000) was used for processing the still images.^13^^,^^14^

A descriptive analysis was made of the results.

## RESULTS

Table 1 shows the amount of HA found in the LP of VFs as measured by Alcian Blue staining. Values are shown according to the VF area and the LP layer that was investigated. These values are percentages of the color area selected by the Imagelab 2000 software compared to the area of the original image.

**Table 1:** Percentage of the color blue in Alcian Blue-stained slides, for the superficial, middle and deep VF layers.

Areas	Superficial Layer	Middle Layer	Deep Layer
1	2,4	23,6	21,7
2	3,1	23,0	33,7
3	1,4	12,0	9,1
4	1,1	9,1	8,9
5	2,0	8,6	9,4
6	1,3	10,0	9,6
7	1,0	10,4	9,3
8	2,9	20,3	18,8
9	1,4	23,5	23,0

**Figure 1:**
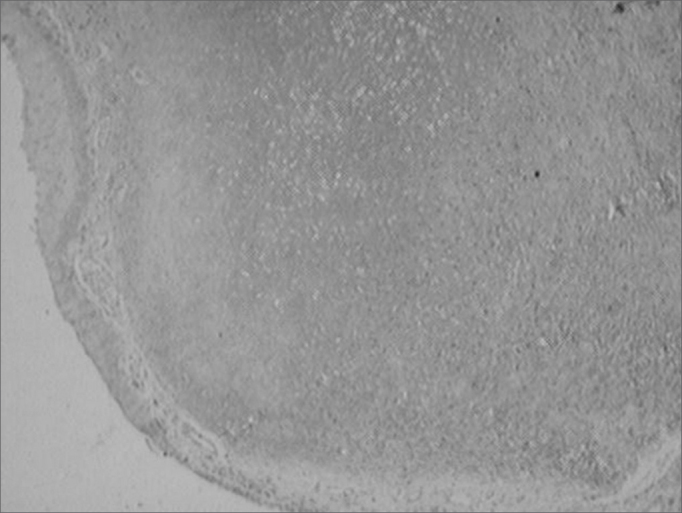
Anterior macula flava - Alcian Blue stained slide

**Figure 2:**
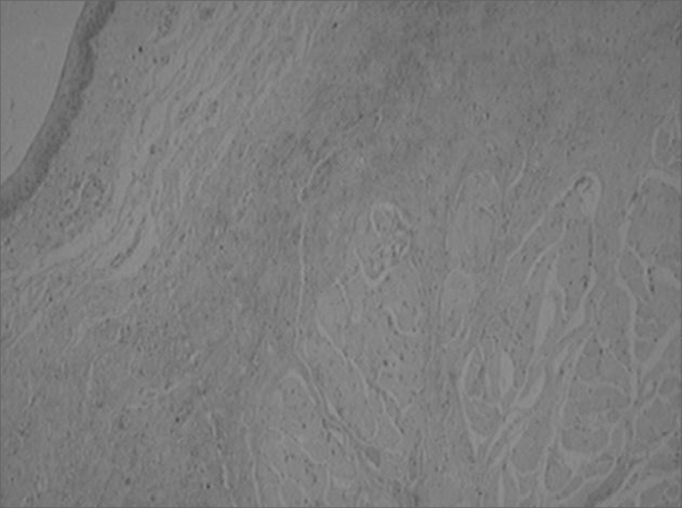
Middle VF area - Alcian Blue stained slide.

**Figure 3:**
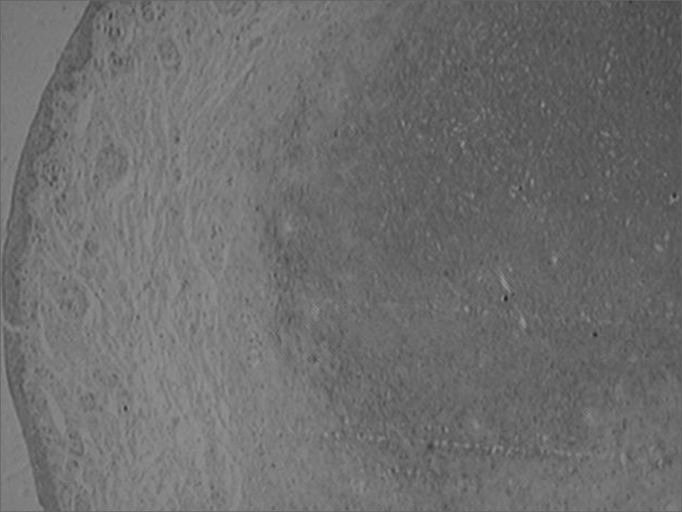
Posterior macula flava - Alcian Blue stained slide.

Values for the middle and deep VF layers were higher compared to those of the superficial layer. This distribution was seen across the VF; lower values were seen in the central VF area, relative to the anterior and posterior areas, in which the maculae flavae are located (Figure 1, Figure 2, Figure 3).

**Figure 4:**
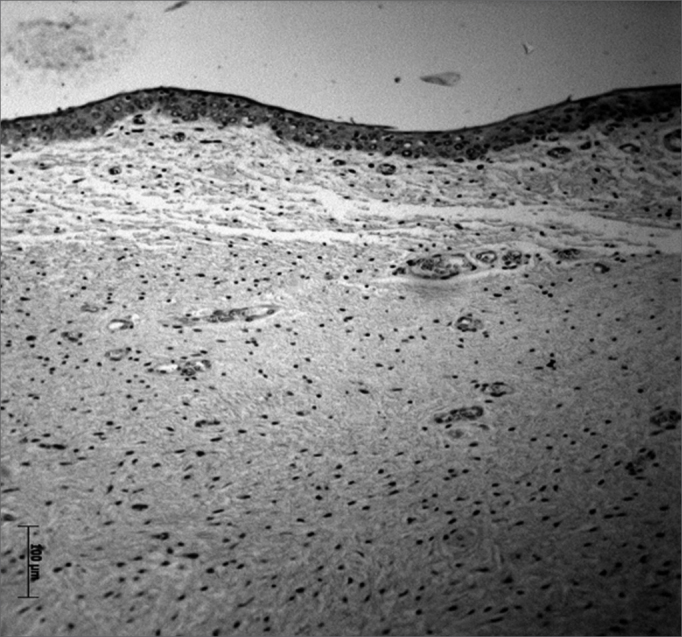
Area of the posterior macula flava - Immunohistochemical method showing absence of epithelial cells (which are stained in brown).

**Figure 5:**
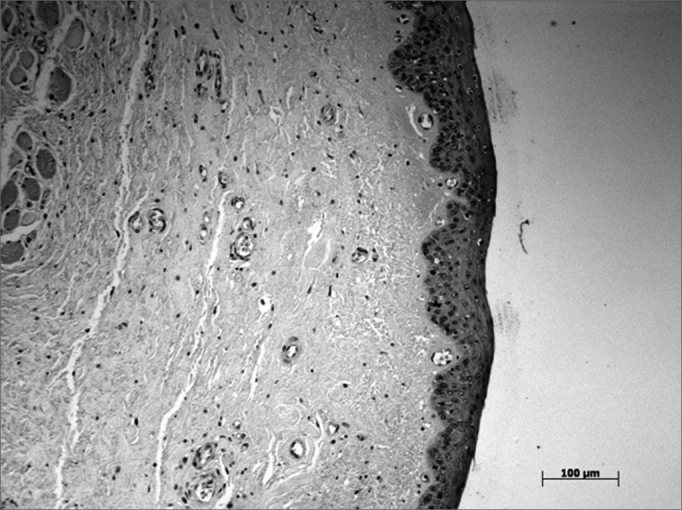
Middle VF area - Immunohistochemical method showing multiple epithelial cells stained brown.

**Figure 6:**
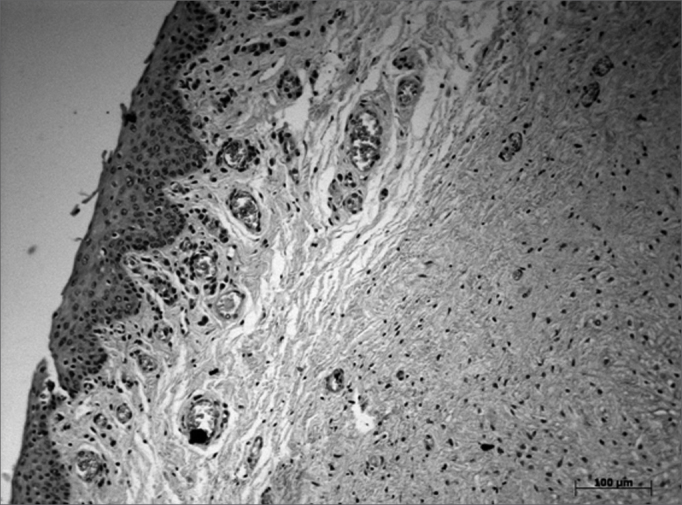
Area of the anterior macula flava - Immunohistochemical method showing few epithelial cells, stained in brown.

Immunohistochemical findings revealed HA receptors in the VF lining epithelium, concentrated mostly in the central region of VFs (areas 4, 5 and 6); a few cells were also found in other areas of the epithelium. Receptors were absent in the LP layers of VFs (Figure 4, Figure 5, Figure 6).

## DISCUSSION

VFs are complex trilaminar structures composed of epithelium, connective tissue, striate muscle, nerves and blood vessels.^15^

The importance of the LP in voice production may be appreciated in clinical practice when anatomical changes and secondary injury affect LP vibration, resulting in dysphonia.^16^

HA and other macromolecules found in the ECM of the LP are important for the mechanical and biological properties of VFs.

Among its functions, HA decreases the dynamic viscosity, which makes it possible to produce sound at high frequencies at a lower energy threshold.^8^

Knowing the distribution and concentration of HA across VFs is necessary for understanding its physiology. Most of the studies so far have analyzed only the middle third of the membranous portion of VFs.

Studies on the concentration and distribution of HA, done by using an HA-binding probe isolated from bovine nasal cartilage as a marker, have revealed that HA is present at higher concentrations in the anterior and posterior maculae flavae, in the middle layer of VFs, around the muscle fibers and in the capsule of glands.^9^^,^^17^

Maculae flavae are composed of elastic fiber bundles, and are an extension of the middle layer of the LP of VFs. These structures protect the vocal ligament from mechanical injury by acting as shock absorbers of impact resulting from VF vibration. It was later found that maculae flavae were composed of collagen fibers, fibroblasts and other extra-cellular substances, and that they had an important role in VF growth and development. Maculae flavae appear to form collagen and elastic fibers, and other ECM components, supporting HA function.^15^

HA is present around vocal muscle fibers for lubrication purpose, facilitating rapid movements. These movements are necessary for the larynx to protect the lower airways during swallowing.^9^

The HA receptor belongs to a group of plasmatic membrane proteins that supports interactions between the cell surface and ECM components. Other members of this group are the fibronectin, collagen, laminin and heparan sulphate receptors. The combined effect of these components gives rise to the cell's adhesive properties.^10^

There is evidence of HA receptor expression in proliferating cells, such as those found in the crypts of Lieberkuhn along the colonic wall, as well as in stratified epithelium such as the tongue, the esophagus and the epidermis of hamsters. HA receptors have not been found in other types of epithelium such as corneal and stomach mesothelial cells.^10^

Studies of 3T3 cells in which growth was inhibited by contact with other cells in high cell density populations revealed that HA receptors were highly expressed in growing cells, and were less expressed in cell groups with no growth.^10^

The relation between CD44 expression and the absence of HA in the formation of pillous follicles suggests, but does not prove, that CD44 expression causes mesenchimal cells and macrophages to degrade HA.^12^

In our study we did not present the specific reactions for HA; however, histology with Alcian Blue yielded similar results to those found by using a technique specific for detecting GAG. Immunohistochemical HA receptor findings were similar to other studies using a similar technique, although we investigated only a single VF.^9^, ^10^, ^11^, ^12^^,^^17^

It appears that a higher concentration of HA receptors in the central area of VFs occurs due to increased local cell proliferation, which in turn takes place because of the increased level of contact between VFs.

The inversely proportional concentration of HA and its receptor along VFs has been reported in other studies. The macula flava contains more HA, which acts as a cushion for absorbing impact, given its hydrophilic properties; there is, consequently, a lower concentration of receptors in this area, which facilitates the penetration of components to the LP of VFs.

Studies on the physiology and the components of the LP of VFs are important in the treatment of minimal structural alterations, which surgery is not able to correct. The disorganization of amorphous structures of the LP remains even after surgery, which causes altered vibrations of the mucous wave to persist.

Understanding the composition and function of the ECM across the various VF layers is necessary for developing novel future therapy.

## CONCLUSION

The immunohistochemical technique makes it possible to assess the distribution of HA receptors in human VFs.
